# Maxillary Tuberosity Reconstruction with Transport Distraction Osteogenesis

**DOI:** 10.1155/2012/816572

**Published:** 2012-05-31

**Authors:** F. Ugurlu, B. Basel, B. Cem Sener, A. Sertgöz

**Affiliations:** ^1^Department of Oral and Maxillofacial Surgery, Faculty of Dentistry, Marmara University Nişantaşı Kampuşı, Büyük Çiftlik Sokak No. 6, Nişantaşı, Şişli, 34365 Istanbul, Turkey; ^2^Department of Prosthodontics, Faculty of Dentistry, Marmara University, 34365 Istanbul, Turkey

## Abstract

Severe bone loss due to pathology in the maxillary tuberosity region is a challenging problem both surgically and prosthetically. Large bone grafts have a poor survival rate due to the delicate bony architecture in this area and presence of the maxillary sinus. Our case presentation describes a new technique for reconstructing severe bony defect in the maxillary tuberosity with horizontal distraction osteogenesis in a 45-year-old man. A 4 × 6 × 3 cm cyst was discovered in the left maxillary molar region and enucleated. Three months postoperatively, the area had a severe bone defect extending to the zygomatic buttress superiorly and hamular notch posteriorly. Three months later, a bone segment including the right upper second premolar was osteotomised and distracted horizontally. The bone segment was distracted 15 mm distally. After consolidation, implants were placed when the distractor was removed. A fixed denture was loaded over the implants after 3 months. Complete alveolar bone loss extending to the cranial base can be reconstructed with transport distraction osteogenesis. Distalisation of the alveolar bone segment adjacent to the bony defect is an easy method for reconstructing such severe defects.

## 1. Introduction

Severe bone loss due to pathology in the maxillary tuberosity region is a challenging problem for aesthetic and functional reconstruction, both surgically and prosthetically [1]. Large bone grafts have a poor survival rate due to the delicate bony architecture in this area and presence of the maxillary sinus [2]. Distraction osteogenesis (DO), which is an alternative approach to such defects, is a biological process that generates new bone with gradual traction of the divided bone segments [3]. The greatest advantage of DO is that the soft tissues expand with the bone during the process, obviating the need for bone grafts and avoiding donor site morbidity.

 Costantino and coauthors first used bifocal distraction to reconstruct a segmental defect in the canine mandible [4]. Gantous and coauthors demonstrated that bone healing was feasible in the irradiated mandible using transport distraction [5]. A large symphyseal defect following a mandibulectomy was treated successfully by applying trifocal distraction to bridge the defect by moving two transport segments toward each other in dogs [6]. Subsequently, this technique was applied in clinical mandibulectomy reconstruction [7–9].

 In the maxilla, Liou and coauthors used transport distraction to close alveolar cleft defects [10]. The osteotomised dental segment was transported forward to obliterate the alveolar space without the need for further bone grafts [10]. In a monkey study, Cheung and coauthors demonstrated that reconstruction of a posterior maxillectomy defect using transport DO is feasible, with bone regeneration in the distraction gap formed by intramembranous ossification, and teeth in the transport segment remain viable [11, 12]. Active bone mineralisation and remodelling occur in the new bone within 3 months after distraction in maxillary dentoalveolar tissue [3, 13].

 This case study describes the clinical management of a patient using transport distraction in posterior maxilla reconstruction and prosthetic rehabilitation after implant placement.

## 2. Case Report

**Figure 1:**
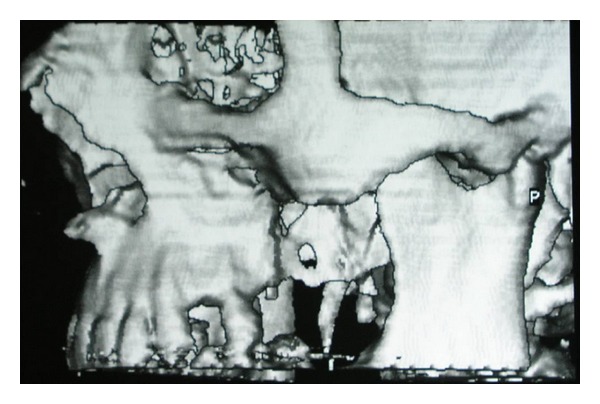
Ct scan view.

**Figure 2:**
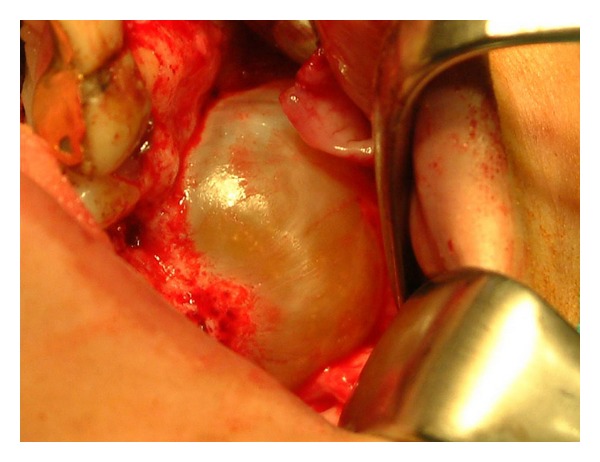
Intraoral view of cyst.

**Figure 3:**
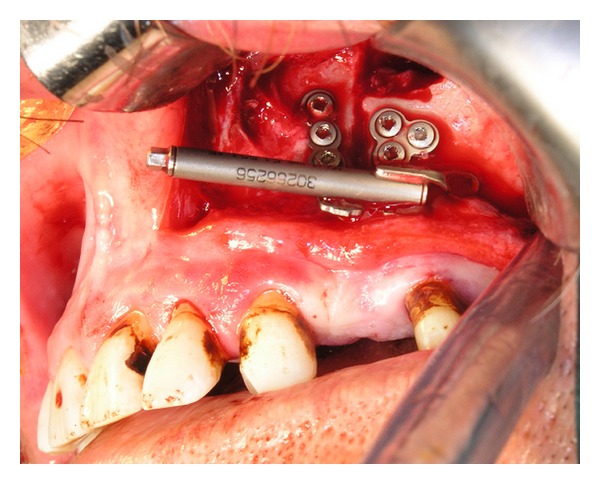
Intraoral view of distractor.

A 45-year-old man, who suffered from pain in the left posterior maxilla and a bad smell from the nose, was referred to our department. A 4 × 6 × 3 cm cyst was found in the left maxillary molar region on radiological examination (Figure 1). After treating the acute infection, the patient underwent cyst enucleation under general anaesthesia (Figure 2). Six months postoperatively, the area had a severe bony defect extending to the zygomatic buttress superiorly and hamular notch posteriorly. After computed tomography (CT) and model analysis of the defect, we decided to reconstruct it using transport distraction. Under general anaesthesia, a vestibular incision was made and a mucoperiosteal flap was raised to expose the lateral wall of the maxilla. The bone between the number 23 and the number 25 maxillary teeth was cut vertically with a saw and then connected to a horizontal bone cut 5 mm above the apex of the second premolar running posteriorly on the buccal side. The bone on the palatinal side was cut horizontally with curved osteotomes, gently to avoid damaging the palatal mucosa. Before mobilising the transport segment including number 25, the distractor was adapted to its stabilising plates with screws, and then the segment was mobilised using osteotomes (Figure 3). Before suturing the surgical site, the distractor was checked to ensure that the transport segment was being moved into the proper position.

**Figure 4:**
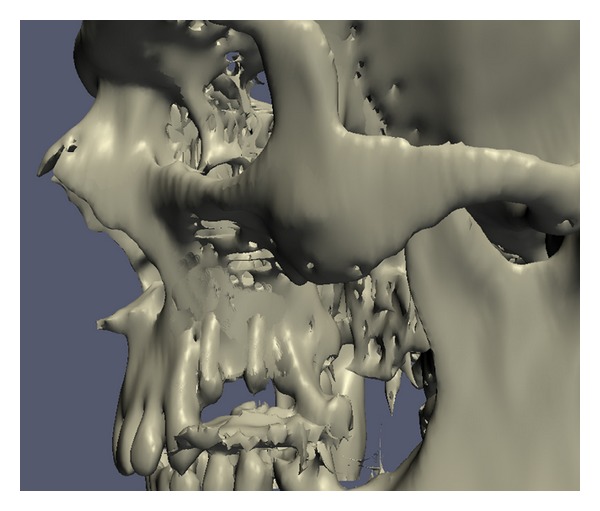
CT scan view after implant placement.

 After a 7-day healing period, the distractor was activated by 1 mm once daily for 15 days. After full activation, the distractor was left *in situ* for the consolidation phase. Six weeks later, the distractor was removed and two dental implants were placed in the new bone. After 3 months for osseointegration, we realised that the implants were not aligned on the proper axis and position on CT (Figure 4). To correct this situation, a subapical osteotomy including the implants was performed and a block autogenous graft was taken from the mandibular symphysis and adapted to the superior border of the osteotomy line.

**Figure 5:**
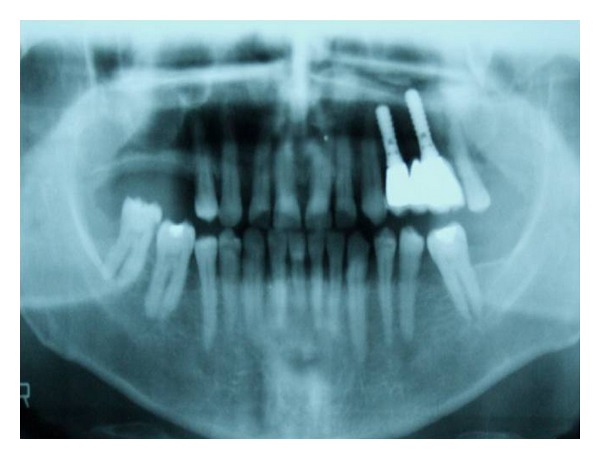
Radiological view after prosthetic rehabilitation.

**Figure 6:**
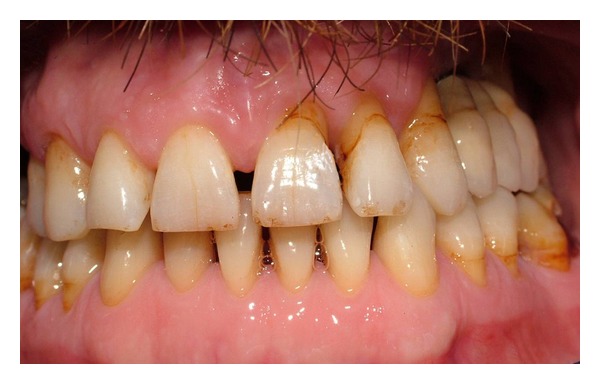
Intraoral view after prosthetic rehabilitation.

 Permanent prosthetic rehabilitation was started 4 months after the subapical osteotomy. Metal-supported porcelain restorations were constructed using conventional methods. The patient was followed up 3, 6, 12 and 24 months after the prosthetic rehabilitation (Figures 5 and 6).

## 3. Discussion

Callus distraction has become a widely established surgical technique, offering excellent results in every body part. For huge jaw defects after resection, cyst enucleation, or trauma, transport distraction osteogenesis (TDO) is a suitable alternative to complex conventional augmentation techniques with either free or microvascular bone grafts. Conventional techniques cause donor site morbidity when harvesting the soft tissues and bone grafts. Furthermore, free bone grafts are commonly associated with unpredictable resorption during healing [11]. The advantages of TDO lie in avoiding these problems, faster wound healing, and shortened hospitalisation. Cheung and coauthors proved the feasibility of TDO as a reconstruction alternative [11, 12]. It restores the alveolar ridge and occludes the oroantral connection readily in one stage without requiring soft tissue and bone grafts, as in our case. The replacement with native dentoalveolar bone obviates the need for a secondary sulcoplasty. The presence of native keratinised attached gingiva in the reconstructed defect and distraction gap is important for maintaining the hygiene of future dental implants, which cannot be achieved with conventional techniques.

 Nevertheless, DO has possible surgical complications during distraction and in the consolidation phase, such as fractures of the transport and anchorage segment, premature segment consolidation, an undesirable transport vector, and inadequate bone formation [14]. In addition, patients may not tolerate the distractor, especially extraosseous distractors [15]. Moreover, fibrotic tissue, ulcer formation, and epithelium invagination on the distraction side can be seen.

 In our case, fibrotic tissue developed on the cheek mucosa as a result of rod irritation. To resolve this issue, the distractor was removed and dental implants were placed after a 6-week consolidation period, during the same operation. Epithelial invagination or ulcer formation due to chronic irritation can also be overcome by cutting the rod or preparing soft coverage for the rod.

 Controversy exists regarding the consolidation time and when the implants should be placed. Ilizarov stated that the consolidation time should be at least as long as the distraction time [3]. According to Swennen and coauthors, 6–8 weeks are sufficient for the mandible and 2-3 months for the maxilla [16]. Pensler and coauthors used a 2-day consolidation period for every 1 mm of distraction in children with hypoplastic mandibles [17]. Block and coauthors placed implants in distracted dog mandibles 10 weeks after completing distraction [18, 19]. Laster and coauthors placed implants after a 7–10 days consolidation period in horizontal alveolar distraction osteogenesis [20]. In our case, fibrotic tissue on the cheek mucosa resulted from rod irritation. To resolve the patient's complaint, the distractor was removed and dental implants were placed after a 6-week consolidation period during the same operation.

 In an animal study, Cheung and coauthors found no changes in the periodontal ligament or alveolar bone around the teeth involved in the interdental osteotomy and distraction [11, 12]. With unintentional exposure of the root surfaces using an osteotome, new bone formed from the intact bone interface [21]. Another concern is the development of pathological pulp changes in the teeth, which occur with a subapical osteotomy if the bone cut is less than 5 mm from the root apices [22]. In our case, the integrity of the pulp tissues for teeth number 23 and number 25 was normal after transport distraction and subapical osteotomy, the transport segment was viable, and no permanent damage was induced by TDO.

## 4. Conclusions

In conclusion, complete alveolar bone losses extending to the cranial base can be reconstructed using TDO. Distalisation of the alveolar bone segment adjacent to the bony defect is an easy, successful reconstruction for such severe defects. Successful distraction depends on the stability of the distractor, proper surgical planning, appropriate distraction vector, and patient cooperation.
